# The standardized ileal digestible lysine-to-net energy ratio in the diets of lactating primiparous sows to optimize maternal nitrogen retention is dynamic but does not impact piglet performance

**DOI:** 10.1093/jas/skaf168

**Published:** 2025-05-14

**Authors:** Nicole L Gregory, Lee-Anne Huber

**Affiliations:** Department of Animal Biosciences, University of Guelph, Guelph, ON, Canada; Department of Animal Biosciences, University of Guelph, Guelph, ON, Canada

**Keywords:** lactation, net energy, nitrogen utilization, primiparous sows, standardized ileal digestible lysine

## Abstract

Fifty-five primiparous sows were recruited to evaluate the effect of increasing standardized ileal digestible (**SID**) Lys-to-net energy (**NE**) ratios on primiparous sow maternal nitrogen retention, milk nitrogen output, and piglet growth performance during each week of lactation. Sows were assigned to one of five diets with equally spaced and increasing SID Lys-to-NE ratios between 2.85 and 5.51 g SID Lys/Mcal NE. The intermediate ratios were achieved by blending the two extreme diets in varying proportions using a feeding system with feed blending capabilities and were provided to sows immediately after farrowing and until weaning (day 24 ± 1). Nitrogen (**N**) balances were conducted between days 4 and 7 (week 1), 12 and 15 (week 2), and 20 and 23 (week 3) with total urine and fecal grab sampling occurring during each N balance period. Linear (**LBL**) and quadratic broken-line and polynomial quadratic models were used to determine the optimum dietary Lys-to-NE ratios for maternal N retention in each week of lactation. The Bayesian information criterion was used to assess best fit. Dietary feeding program did not influence sow average daily feed intake in lactation, piglet average daily gain and body weight (**BW**) at weaning, or milk N output during any nitrogen balance period. Sows fed increasing SID Lys-to-NE ratios lost less BW and backfat by the end of lactation (linear; *P* < 0.05). Sow N intake, N excretion, whole-body N retention (N intake − N excretion in urine and feces), and maternal N retention (whole-body N retention − milk N output) increased as dietary SID Lys-to NE ratio increased within each N balance period (linear; *P *< 0.05). The LBL model showed the optimal SID Lys-to-NE ratio for maternal N retention was out of the range of the dietary treatments for week one, but maternal N retention was optimized at 4.74 and 4.85 g SID Lys/Mcal NE in weeks two and three, respectively. Therefore, a dynamic SID Lys-to-NE ratio during each week of lactation could be implemented to enhance maternal N retention of primiparous sows.

## Teaser

Increasing dietary Lys-to-net energy (**NE**) ratio in lactating primiparous sow diets improved maternal nitrogen retention but did not affect piglet growth performance. Incorporating dynamic Lys-to-NE ratios in the diets of primiparous sows could be a means to minimize maternal nitrogen loss during the first lactation.

## Introduction

Milk synthesis requires significant amounts of nutrients and energy, with a two-fold increase in energy and standardized ileal digestible (**SID**) Lys requirements between farrowing and peak lactation and up to 80% of estimated energy requirements attributed to milk production (Feyera and Theil, 2017; as reviewed by Tokach et al., 2019). Since milk is the primary nutrient source for nursing piglets, the ability of a sow to produce adequate milk is essential for piglet growth. As larger litter sizes become more prevalent in commercial sow herds, meeting nutrient and energy requirements is critical for milk production and piglet growth performance.

The appetite of the sow is a limiting factor for milk production in lactation. Low feed intake is especially an issue for primiparous sows that also have reduced gut capacity compared to multiparous sows, further hindering sufficient energy and nutrient intakes to support lactation without excessive maternal tissue (fat and protein) mobilization (Strathe et al., 2017). Moreover, excessive mobilization of maternal tissues can negatively impact future reproductive performance leading to smaller subsequent litter sizes, due to reduced ovulation rates, and inadequate body condition during the next lactation (Clowes et al., 2003; Schenkel et al., 2010; Hoving et al., 2011). Therefore, meeting both energy and amino acid (Lys) requirements throughout lactation is necessary to support milk production as well as future reproductive performance and, consequently, sow longevity.

Feyera and Theil (2017) suggested that the dynamic nature of energy and Lys requirements during lactation are driven by milk production and sow body weight (**BW**), which is contradictory to current feeding practices where sows are fed a single diet throughout lactation and among parities. Thus, we hypothesized that the optimal SID Lys-to-net energy (**NE**) ratio will change throughout lactation for primiparous sows corresponding to milk production. The objective of the study was to determine the SID Lys-to-NE ratios that minimize primiparous sow maternal nitrogen loss and maximize milk nitrogen output and piglet growth performance during each week of lactation.

## Materials and Methods

The study was conducted at the Arkell Swine Research Station (Guelph, ON, Canada). The experimental protocol was approved by the University of Guelph Animal Care Committee (animal utilization protocol 4988) and followed Canadian Council of Animal Care guidelines (CCAC, 2009).

### Animals and housing

Fifty-five primiparous sows (Yorkshire and Yorkshire × Landrace) were recruited over six consecutive farrowing batches (blocks). Sows were moved into individual farrowing crates equipped with a heat mat in the creep area on day 110 ± 2 of gestation. Sows were provided feed via an electronic sow feeder that was capable of blending two diets (Gestal Quattro, JYGA Technologies, St-Lamert-de-Lauzon, Quebec, Canada) and received 2 kg per day of a blended diet delivering 4.18 g SID Lys/Mcal NE until farrowing. Upon farrowing (day 1 of lactation), litters were standardized to 12.3 ± 0.4 piglets. Within 24 h, piglets were processed following farm protocols (ear notching, needle teeth clipping, tail docking, and iron injection) and males were surgically castrated on day five of lactation. Piglets were not provided creep feed to allow for BW gain to reflect milk production. Sows and piglets received ad libitum access to water. Sow BW was measured at farrowing, on days 4, 12, and 20 of lactation, and at weaning (24 ± 1 d). In addition, sow backfat depth (**BF**) measurements were taken at the P2 position of the last rib using a portable ultrasound machine with a 140 mm linear probe (Agroscan L, ECM Noveko International Inc., Angoulême, France) on days 1 and 24 of lactation. Piglets were weighed at birth, on days 4, 7, 12, 15, and 20 of lactation, and at weaning (24 ± 1 d).

### Dietary treatments

Upon farrowing, sows were assigned to one of five dietary treatments considering initial BW and breed (Yorkshire or Yorkshire × Landrace); sows received the dietary treatment for the entire lactation period. The dietary treatments provided equally spaced and increasing SID Lys-to-NE ratios between 2.85 and 5.51 g SID Lys/Mcal NE (Table 1), with titanium dioxide as an indigestible marker to estimate apparent total tract nitrogen (**N**) digestibility. Using the electronic sow feeders, the two extreme isocaloric basal diets were blended in proportions of 75:25, 50:50, and 25:75 to create the intermediate ratios of 3.51, 4.18, and 4.84 g SID Lys/Mcal NE, respectively. The basal diets were formulated to meet or exceed the recommended amino acid (**AA**)-to-Lys ratios to ensure that SID Lys remained as the first limiting AA (NRC, 2012) and the 50:50 blend met the estimated average energy and SID Lys requirements for sows using the NRC (2012) lactating sow model with the inputs: anticipated BW at farrowing of 190 kg, piglet average daily gain (**ADG**) of 230 g/day, and litter size of 13 piglets. Accordingly, the feeding curve provided approximately 4.95 kg of diet blend by day 4, 6.89 kg by day 12, and 7.41 kg by day 20 of lactation. Sows were able to receive feed between 0600 and 2000 via manual trigger manipulation. Between days 4 and 7 ± 1, 12 and 15 ± 1, and 20 and 23 ± 1 of lactation, feed was weighed and blended by hand, and any feed refusals were measured daily to calculate feed intake. Calibration of the feeding system occurred weekly by dispensing 500-g allotments of each basal diet. During calibration, representative subsamples from each basal diet were collected and combined within treatment per block for further analysis.

**Table 1.** Ingredient composition and nutrient content of experimental diets (as-fed)

	SID Lys:NE, g/Mcal^1^	
	2.85	5.51
Ingredients %		
Corn	44.48	33.35
Wheat, soft red	20.00	20.00
Soybeans, full fat	13.50	25.50
Soybean meal	5.00	15.25
Soybean hulls	9.00	0
Fat, animal-vegetable blend	3.00	0.10
Monocalcium phosphate	1.85	1.60
Limestone, ground	1.10	1.15
Premix^2^	0.60	0.60
Sodium chloride	0.50	0.50
L-Lys-HCL	0.22	0.53
L-Val	0.23	0.38
L-Thr	0.17	0.27
L-Leu	0.03	0.20
DL-Met	0.04	0.15
L-Phe	0.06	0.14
Titanium dioxide	0.10	0.10
L-Trp	0.04	0.05
L-His	0.04	0.09
L-Ile	0.04	0.04
Total	100.00	100.00
Calculated nutrient contents^3^		
Net energy, Mcal/kg	2.54	2.54
Gross energy, Mcal/kg	4.18	4.16
Crude protein, %	14.92	23.26
SID Lys, %^4^	0.72	1.40
SID Met, %	0.24	0.44
SID Thr, %	0.57	0.92
SID Leu, %	1.01	1.61
SID Val, %	0.75	1.2
SID Ile, %	0.49	0.79
SID His, %	0.35	0.56
SID Phe, %	0.62	1.02
Total calcium, %	0.91	0.91
STTD P, %^5^	0.52	0.53
Fermentable fiber, %	11.22	11.98
Analyzed nutrient contents^6^		
Gross Energy, kcal/kg	4,147	4,238
Crude protein, %	15.90	23.30
Lys, %	0.85 (0.86)^7^	1.47 (1.59)
Met, %	0.34 (0.28)	0.53 (0.49)
Thr, %	0.73 (0.68)	1.05 (1.08)
Leu, %	1.46 (1.20)	2.02 (1.89)
Val, %	0.96 (0.88)	1.37 (1.38)
Ile, %	0.69 (0.59)	0.99 (0.94)
His, %	0.46 (0.41)	0.78 (0.64)
Phe, %	0.92 (0.73)	1.37 (1.18)

^1^SID lysine-to-NE ratio.

^2^Provided the following amounts of vitamins and minerals per kg of premix: Calcium 22.67% as CaCO_3_; Manganese, 4,000 mg as MnSO_4_ H_2_O; Zinc, 21,000 mg as ZnSO4; Iron, 20,000 mg as FeSO_4_; Copper, 3,000 mg as CuSO4; Selenium, 60 mg as Na_2_SeO_3_; Iodine, 100 mg as C_2_H_10_I_2_H_2_; Vitamin A, 2,000 KIU; Vitamin D, 200 KIU; Vitamin E, 8,000 IU; Vitamin K, 500 mg; Thiamin, 200 mg; Riboflavin, 1,000 mg; Niacin, 5,000 mg; Panthothenic Acid, 3,000 mg; Pyridoxine, 300 mg; Choline, 100,000 mg; Folacin, 400 mg; Biotin, 40 mg; Vitamin B12, 5,000 mcg (Grand Valley Fortifiers Ltd., Cambridge, ON, Canada).

^3^Based on digestible nutrient and NE contents of feed ingredients according to the NRC (2012).

^4^SID.

^5^Standardized total tract digestible.

^6^Analyzed values for a composite sample of three batches per diet.

^7^Calculated values are shown in parentheses.

### Nitrogen balance procedure and blood and milk sampling

Nitrogen balances were conducted in weeks 1 (days 4 to 7 ± 1), 2 (12 to 15 ± 1), and 3 (20 to 23 ± 1) of lactation for each sow within the farrowing crates. Briefly, on the first day of the N balance, a sterile Foley catheter was lubricated and inserted into the bladder, and the balloon was inflated with 55 mL of sterile saline (BARDEX I.C., 2-way, Specialty, Tiemann Model, 75cc balloon, 18FR, Bard Medical, Covington, GA). Polyvinyl tubing was attached to the base of the catheter to allow for total urine to be collected in a covered bucket that contained H_2_SO_4_ to maintain a pH of less than 4. Every 24 h, urine was weighed, and a 5 % subsample (wt/wt) was collected and pooled within sow and N balance period. At the end of the N balance period, the pooled sample was mixed, subsampled, and stored at 4 °C until further analysis. If the catheter became disconnected from the bucket, an additional day was added to the N balance period to ensure three complete days of urine collection. Fresh fecal samples were collected by stimulating the rectum. Fecal samples were collected daily, pooled per sow within N balance period, and stored at −20 °C. At the end of each N balance period, the urinary catheters were removed.

After a 17-h fasting period, blood was collected from sows via an orbital sinus puncture on days 4, 12, and 20 of lactation. Blood was collected into 10-mL serum and 6-mL sodium heparin plasma tubes (BD vacutainer; Franklin lakes, NJ, USA); tubes were centrifuged at 1,500 × *g* for 15 min at 4 °C and serum and plasma were subsampled and stored at −20 °C until further analysis.

On the final day of each N balance period, piglets were removed from the sows for 1 h and 1 mL of oxytocin [Oxyto-Sure (20 USP/mL), Vetoquinol, QC, Canada] was injected intramuscularly to ensure milk let down. Approximately 75 mL of milk were collected each from an anterior and posterior gland until glands were emptied, pooled per sow, and stored at −20 °C until further analysis.

### Sample analysis

Feed samples from each block were pooled and a representative subsample was used for chemical analysis. Fecal samples were freeze-dried for 6 d and then finely ground prior to analysis. Feed and freeze-dried fecal samples were analyzed for dry matter by drying for 3 h at 100 °C (AOAC, 2005; method 930.1) and then were ashed in a 600 °C furnace for 12 h (AOAC, 2005; method 942.05). Titanium dioxide contents in the ashed feed (quadruplicate) and fecal (duplicate) samples were measured using spectrophotometry at 407 nm following the procedure described by Crosbie et al. (2020). Milk samples were thawed, mixed, and a 30-mL subsample was freeze-dried for 72 h to determine dry matter content (duplicate). Freeze-dried milk samples were then used to determine crude fat via high-temperature solvent extraction (duplicate; Ankom, XT29 Fat Analyzer, Macedon, NY; AOCS, 2017; Official Procedure Am 5-04). Nitrogen contents in feed, feces, urine, and milk were analyzed via combustion (singlicate; LECO-FP 828 analyzer, LECO Instruments Ltd., Mississauga, ON, Canada). Nitrogen values were multiplied by 6.25 for feed and by 6.38 for milk to calculate crude protein (**CP**) contents.

Plasma AA was analyzed via ultra-performance liquid chromatography (Waters Corporation, Milford, MA) adapted from Bidlingmeyer et al. (1984), as described by Banton et al. (2021). The acid hydrolysis method was used for all AA, except for Met and Cys that used performic acid oxidation with acid hydrolysis (Method 994.12; AOAC, 2005; Waters Corporation, Milford, MA). Derivatization was completed with the AccQ-Tag Ultra derivatization kit (Waters Corporation, Milford, MA). Peaks for AA were compared to the peaks of known standards, and areas were quantified using Waters Empower 2 Software (Waters Corporation, Milford, MA). Feed AA content was analyzed similarly but using oxidative hydrolysis.

### Calculations and statistical analysis

Nitrogen intake was determined by multiplying daily feed intake and analyzed N contents of the basal diets, using weighted averages of the basal diets N contents for the intermediate ratio blends. Fecal N excretion was determined using N intake and apparent total tract N digestibility (Zhu et al., 2005). Whole-body N retention was calculated by subtracting N excreted in urine and feces from N intake (Möhn and de Lange, 1998). Milk N output was determined using the N content in the milk and estimated daily milk production (NRC, 2012; equation 8-71) during the corresponding N balance period. Maternal N retention was calculated by subtracting milk N output from whole-body N retention. The apparent AA utilization efficiencies for milk production were calculated as described by Huber et al. (2016).

All statistical analyses were conducted using the Proc GLIMMIX function of SAS 9.4 with sow (or litter) as the experimental unit. The model included dietary treatment as the fixed effect and the random effect of block, with the covariant of breed used in the model for sow performance outcomes. Contrast statements were used to assess linear and quadratic effects of increasing Lys-to-NE ratios. Probability (***P***) values less than 0.05 were deemed statistically significant, while 0.05 ≤ *P* ≤ 0.10 were considered a trend. The broken-line linear, broken-line quadratic, and quadratic polynomial models were used as described in Gonçalves et al. (2016); milk N output and litter growth performance were not influenced by SID Lys-to-NE ratio so optimizations were not completed. The Bayesian information criteria were used to assess the model with the best fit, which was then used to determine the ideal ratio of SID Lys-to-NE for maternal N retention in each week of lactation.

## Results

Analyzed AA values for the basal diets (2.85 and 5.51 g SID Lys/Mcal NE) were generally 10% to 20% greater than calculated, but analyzed Lys was comparable to calculated contents. The analyzed gross energy content was comparable between the basal diets. Two sows were removed from the study due to poor feed intake and high BW loss (one each from 4.18 and 4.84 g SID Lys/Mcal NE) and were not included in the statistical analysis. An additional sow completed two nitrogen balance periods before being removed due to a sudden reduction in feed intake; data from the sow was included in the statistical analyses for the first two nitrogen balances, but not the third.

Initial sow BW and BF depth did not differ among dietary treatments (Table 2). Sow BW loss between days 4 and 12 and between days 12 and 20 decreased with increasing SID Lys-to-NE ratio (linear; *P* < 0.05) but was not influenced by dietary treatment between days 20 and 24 of lactation. Sow BW and BF loss over the entire lactation period decreased with increasing SID Lys-to-NE ratio (linear; *P *< 0.01) but average daily feed intake was not influenced by dietary treatment. Litter size at farrowing or at weaning, initial piglet BW, final piglet BW, or overall piglet ADG were not influenced by dietary treatment.

**Table 2.** Sow and litter growth performance over a 24-d lactation for primiparous sows fed one of five isoenergetic feeding programs that provided equally spaced and increasing SID Lys-to-NE ratios

	Diet^1^					SEM^2^	*P*-value^3^	
Item	2.85	3.51	4.18	4.84	5.51		Linear	Quadratic
No. of sows	11	10	10	10	11			
Initial sow BW, kg^4^	181.2	185.2	183.8	180.6	186.3	4.8	0.674	0.888
Sow BW change days 4 to 12, kg	−9.5	−7.7	−7.8	−3.7	−4.6	2.4	0.017	0.822
Sow BW change days 12 to 20, kg	−5.8	−0.6	−3.6	0.1	−4.1	1.8	0.021	0.795
Sow BW change days 20 to 24, kg	0.9	1.0	0.7	3.5	2.3	2.0	0.348	0.948
Total Sow BW change, kg	−19.0	−13.0	−15.7	−5.9	−9.0	3.7	0.001	0.575
Initial sow back fat depth, mm^4^	16.9	14.9	16.0	15.5	16.0	0.8	0.697	0.316
Change in sow back fat depth, mm	−3.9	−4.3	−3.0	−2.0	−2.3	0.7	0.003	0.891
Litter size after standardization^5^	13	12	13	12	12	0.4	0.210	0.922
Litter size at weaning	11	11	12	11	12	0.4	0.955	0.686
Initial average piglet BW, kg^6^	1.37	1.36	1.33	1.41	1.36	0.1	0.915	0.934
Final average piglet BW, kg	6.01	6.57	6.32	6.46	6.31	0.24	0.526	0.273
Piglet average daily gain, g	206	230	217	220	224	10	0.439	0.566
ADFI, kg, as-fed^7^	5.1	5.6	5.2	5.3	5.3	0.2	0.699	0.558

^1^Increasing SID Lys-to-NE ratios, g/Mcal.

^2^Largest value for standard error of the means.

^3^Probability values for linear and quadratic contrasts.

^4^Measurement taken immediately after farrowing.

^5^Standardization of litters occurred within 24 h after birth.

^6^Average piglet BW after standardization.

^7^Sow average daily feed intake.

In weeks 1 and 2 of lactation, estimated SID Lys intake, N intake, total and urinary N excretion, urine weight produced, N absorbed, N retention, and maternal N retention increased with increasing SID Lys-to-NE ratio (linear; *P *< 0.05; Table 3). Fecal N excretion tended to increase with increasing SID Lys-to-NE ratio (linear; *P *= 0.059 and *P *= 0.077 in weeks one and two, respectively). Total milk N output and apparent N retention efficiencies were not influenced by dietary treatment in weeks one or two. In week three of lactation, estimated SID Lys intake, N intake, N excretion, urine weight produced, N absorbed, N retention, and maternal N retention increased with increasing SID Lys-to-NE ratio (linear; *P* < 0.01), while apparent N retention efficiencies decreased with increasing SID Lys-to-NE ratio (linear; *P *< 0.05). Only total milk N output was not influenced by dietary treatment in week three. Milk chemical composition and estimated yield were not influenced by dietary treatment on days 7, 15, or 23 of lactation (Table 4). The exceptions were for crude fat on day 7 that tended to decrease (linear; *P *= 0.083) and CP on day 15 that tended to increase (linear; *P *= 0.095) with increasing SID Lys-to-NE ratio.

**Table 3.** Nitrogen utilization between days 4 and 7 ± 1 (week 1), days 12 and 15 ± 1 (week 2), and days 20 and 23 ± 1 (week 3) of lactation in primiparous sows fed one of five isoenergetic feeding programs that provided equally spaced and increasing SID Lys-to-NE ratios

	Diet^1^					SEM^2^	*P*-value^3^	
Item	2.85	3.51	4.18	4.84	5.51		Linear	Quadratic
**Week 1 (days 4 to 7 ± 1)**								
No. of sows	9	11	10	11	11			
Estimated SID Lys intake, g/d	26.2	35.5	34.1	45.7	51.2	2.1	<0.001	0.453
Feed intake, kg/d, DM	3.5	4.0	3.4	4.1	4.1	0.2	0.041	0.786
N intake, g/d	78.1	102.2	95.8	125.2	136.9	6.2	<0.001	0.601
Total N excretion, g/d	35.0	46.6	47.2	63.4	61.2	4.3	<0.001	0.381
Fecal N, g/d	12.7	16.4	15.3	16.0	17.0	1.8	0.059	0.462
Urinary N, g/d	22.0	29.3	31.7	47.7	44.9	3.5	<0.001	0.551
Urine weight, kg/d	3.7	5.8	6.8	8.1	7.2	0.9	0.001	0.060
N absorbed, g/d^4^	65.2	85.1	80.4	108.9	120.0	5.8	<0.001	0.399
N retention, g/d^5^	43.4	56.6	48.9	63.2	76.3	5.6	<0.001	0.276
Total milk N, g/d^6^	66.0	74.8	64.0	69.3	72.2	4.6	0.653	0.771
Maternal N retention, g/d^7^	-22.8	-18.7	-15.1	-6.0	4.2	6.5	0.021	0.468
N retained, % of intake	54.6	54.6	49.8	49.1	55.6	3.4	0.761	0.166
N retained, % of absorbed	65.5	65.8	59.6	55.9	62.9	3.7	0.184	0.204
**Week 2 (days 12 to 15 ± 1)**								
No. of sows	10	8	11	10	11			
Estimated SID Lys intake, g/d	33.4	44.9	47.8	53.6	63.9	2.4	<0.001	0.863
Feed intake, kg/d, DM	5.2	6.0	5.5	5.6	5.8	0.3	0.111	0.414
N intake, g/d	117.5	152.4	154.5	173.5	192.7	6.9	<0.001	0.522
Total N excretion, g/d	44.3	56.5	55.6	70.7	76.5	6.0	<0.001	0.833
Fecal N, g/d	20.3	24.2	20.9	24.4	23.6	1.3	0.077	0.620
Urinary N, g/d	24.1	32.7	34.8	46.3	52.6	5.4	<0.001	0.751
Urine weight, kg/d	4.8	6.2	7.9	10.3	9.4	1.4	<0.001	0.312
N absorbed, g/d^5^	97.7	129.5	134.0	148.2	168.1	6.2	<0.001	0.477
N retention, g/d^6^	73.6	96.4	99.2	102.1	115.9	6.6	<0.001	0.391
Total milk N, g/d	69.5	78.1	74.3	69.3	79.9	6.6	0.476	0.876
Maternal N retention, g/d^7^	3.6	19.0	24.6	35.2	36.3	8.4	0.001	0.371
N retained, % of intake	61.9	62.4	64.1	58.5	59.6	3.2	0.345	0.511
N retained, % of absorbed	75.0	74.3	74.4	69.1	69.0	3.4	0.079	0.686
**Week 3 (days 20 to 23 ± 1)**								
No. of sows	10	9	10	10	10			
Estimated SID Lys intake, g/d	40.1	48.2	55.0	65.4	68.2	1.4	<0.001	0.169
Feed intake, kg/d, DM	6.3	6.5	6.3	6.6	6.3	0.2	0.466	0.274
N intake, g/d	142.9	165.4	178.8	203.7	210.4	4.7	<0.001	0.164
Total N excretion, g/d	53.0	61.2	64.1	80.7	88.9	4.1	<0.001	0.323
Fecal N, g/d	24.7	25.8	25.2	30.3	27.9	1.6	0.041	0.816
Urinary N, g/d	28.2	35.1	39.1	51.3	61.5	4.1	<0.001	0.294
Urine weight, kg/d	5.5	6.9	9.6	10.7	10.6	1.5	<0.001	0.221
N absorbed, g/d^4^	118.1	139.6	154.0	174.6	182.9	4.3	<0.001	0.107
N retention, g/d^5^	89.8	103.9	114.2	124.0	121.8	4.7	<0.001	0.054
Total milk N, g/d^6^	80.8	89.8	84.9	85.4	87.0	6.8	0.670	0.692
Maternal N retention, g/d^7^	9.0	14.0	29.8	38.0	34.8	6.8	<0.001	0.267
N retained, % of intake	62.8	63.0	64.1	60.5	57.7	1.9	0.029	0.115
N retained, % of absorbed	76.1	75.0	74.6	70.9	66.1	2.6	0.005	0.208

^1^Increasing SID Lys-to-NE ratios, g/Mcal.

^2^Largest value for standard error of means.

^3^Probability values for linear and quadratic contrasts.

^4^N intake − N excreted in feces.

^5^N intake − N excreted in feces − N excreted in urine.

^6^Calculated using litter size and piglet average daily gain between days 4 and 7 for week 1, days 12 and 15 for week 2, and days 20 and 23 for week 3 and analyzed milk N concentrations on days 7, 15, and 21, respectively.

^7^N retention − total milk N output.

**Table 4.** Milk composition for primiparous sows fed one of five isoenergetic feeding programs that provided equally spaced and increasing SID Lys-to-NE ratios

	Diet^1^					SEM^2^	*P*-value^3^	
Item	2.85	3.51	4.18	4.84	5.51		Linear	Quadratic
**Day 7**								
No. of sows	11	10	11	11	9			
Dry matter, %	20.7	21.8	21.1	21.1	20.8	0.6	0.752	0.289
Crude fat, %	10.8	10.4	9.5	9.6	9.5	0.7	0.083	0.399
Crude protein, %^4^	5.3	5.3	5.2	5.4	5.3	0.2	0.931	0.784
Estimated milk yield, kg/day	7.27	8.87	7.98	8.07	8.73	0.61	0.284	0.694
**Day 15**								
No. of sows	11	9	11	11	11			
Dry matter, %	19.6	20.3	19.7	20.4	20.1	0.6	0.495	0.753
Crude fat, %	8.4	8.5	8.0	9.2	9.0	0.6	0.214	0.532
Crude protein, %	4.9	5.0	5.3	5.2	5.3	0.2	0.095	0.750
Estimated milk yield, kg/day	8.81	9.88	9.22	8.02	9.39	0.73	0.689	0.911
**Day 23**								
No. of sows	10	10	10	10	11			
Dry matter, %	19.4	20.1	19.4	19.7	19.8	0.5	0.730	0.867
Crude fat, %	8.0	8.9	7.8	7.4	8.5	0.5	0.688	0.561
Crude protein, %	5.4	5.4	5.4	5.6	5.7	0.2	0.223	0.518
Estimated milk yield, kg/day	9.33	10.55	10.02	9.81	9.24	0.66	0.519	0.150

^1^Increasing SID Lys-to-NE ratios, g/Mcal.

^2^Largest value for standard error of means.

^3^Probability values for linear and quadratic contrasts.

^4^Milk crude protein was calculated as milk *N* × 6.38.

**Figure 1.**
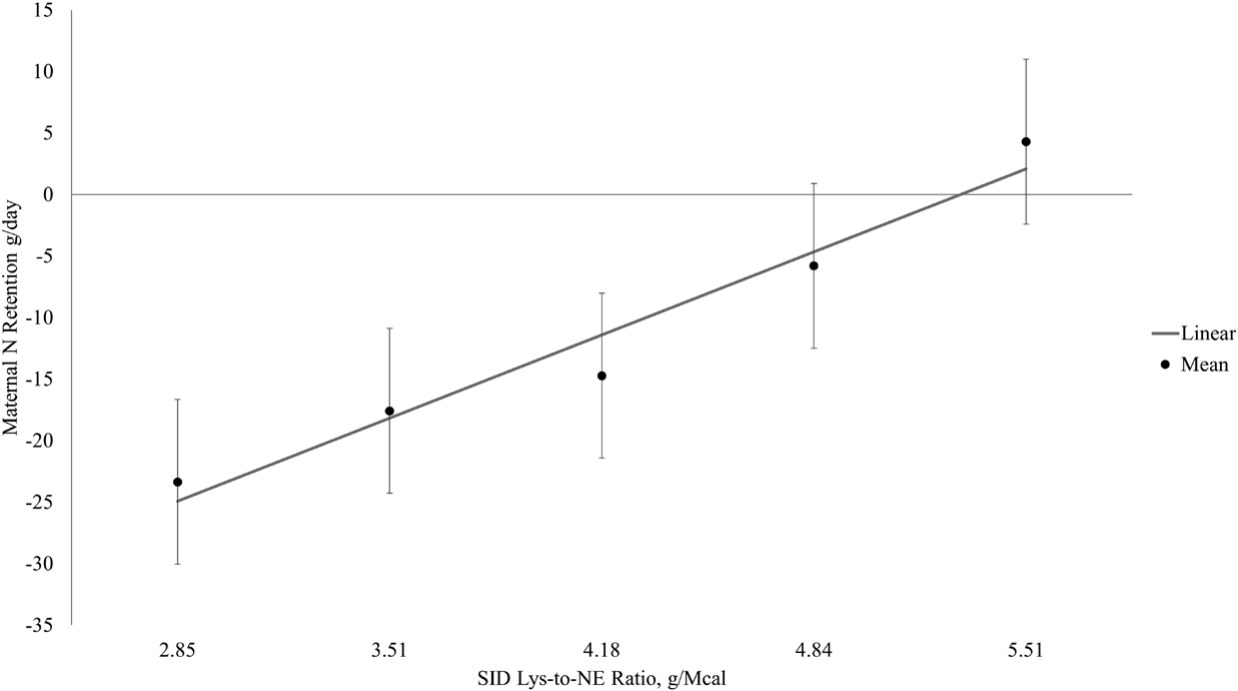
Maternal nitrogen retention between days 4 to 7 of lactation (week 1). Data were best described by the broken-line linear model, but the optimum SID Lys-to-NE ratio was outside of the treatment range.

**Figure 2.**
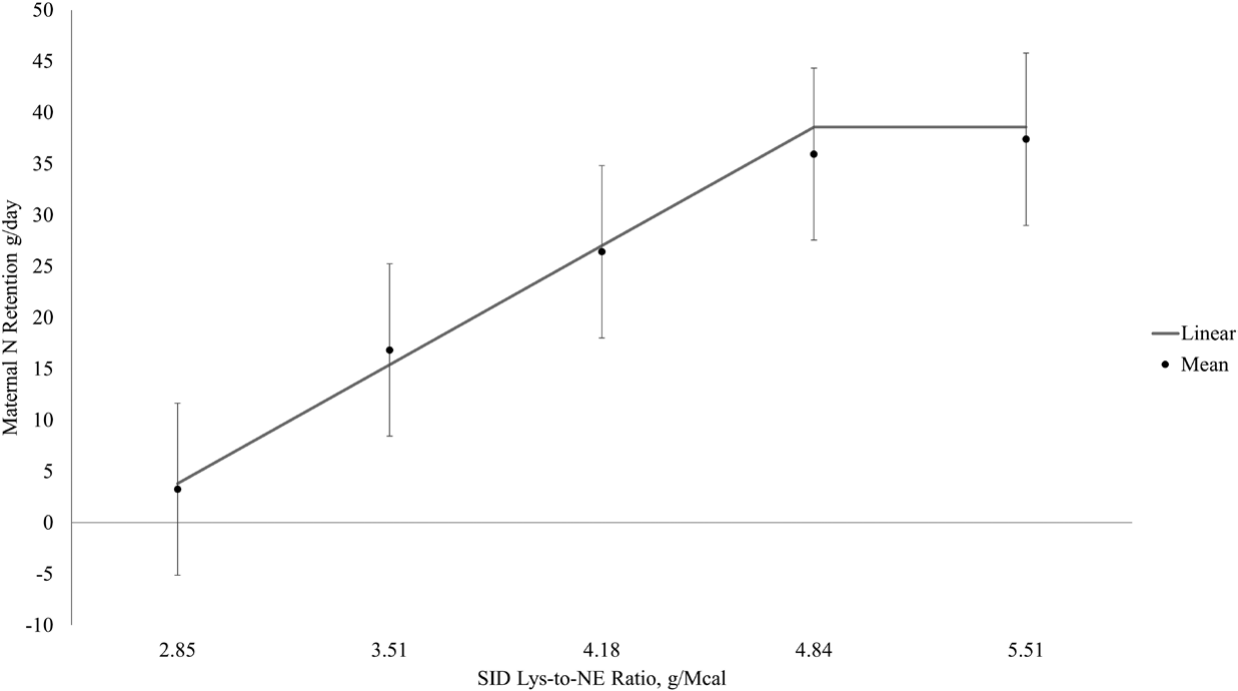
Maternal nitrogen retention between days 12 to 15 of lactation (week 2). Data were best described by the broken-line linear model, and the optimum SID Lys-to-NE ratio was identified at 4.73 g SID Lys/Mcal NE. Maternal N retention, g/d = 36.71 + 17.41*(4.73-SID Lys-to-NE) if SID Lys-to-NE ratio < 4.73.

**Figure 3.**
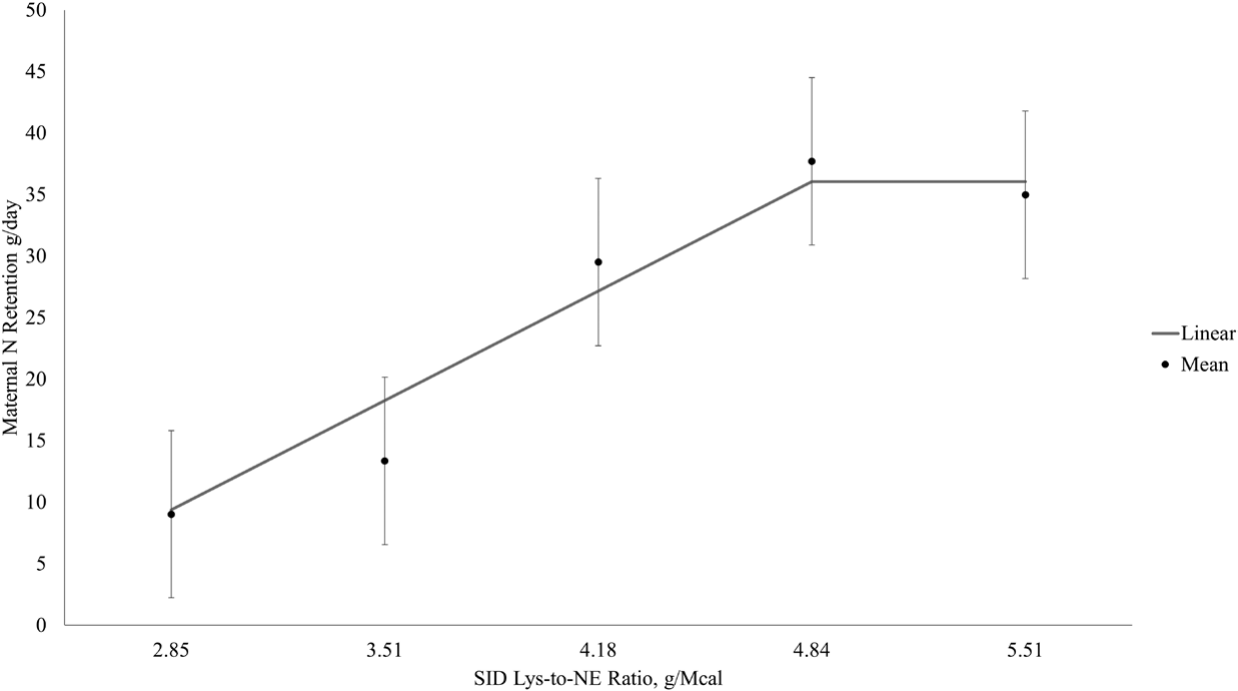
Maternal nitrogen retention between days 20 to 23 of lactation (week 3). Data were best described by the broken-line linear model, and the optimum SID Lys-to-NE ratio was identified at 4.85 g SID Lys/Mcal NE. Maternal N retention, g/d = 36.10 + 13.35*(4.85-SID Lys-to-NE) if SID Lys-to-NE ratio < 4.85.

The broken-line linear model had the best fit for maternal N retention. In week one of lactation, the optimum SID Lys-to-NE ratio was beyond the treatment range (Figure 1) but optimum SID Lys-to-NE ratios were identified at 4.74 and 4.85 g Lys/Mcal NE for weeks 2 and 3 of lactation, respectively (Figures 2 and 3, respectively), which based on the ADFI for week two and three corresponded to SID Lys intakes of approximately 68 and 79 g/d, respectively.

On day 4 of lactation, plasma concentrations of His, Thr, Ala, Asn, Gln, Glu, Gly, and total nonessential amino acids (**NEAA**) decreased with increasing SID Lys-to-NE ratio (linear; *P* < 0.05), whereas plasma Val tended to increase with increasing SID Lys-to-NE ratio (linear; *P* = 0.074; Table 5). On day 12 of lactation, plasma concentrations of His, Ala, Asn, Asp, Gln, Glu, Ser, and NEAA decreased with increasing SID Lys-to-NE ratio (linear; *P* < 0.05). On day 20 of lactation, plasma concentrations of His, Asn, Gln, Gly, Ser, and total NEAA decreased with increasing dietary SID Lys-to-NE ratio (linear; *P* < 0.05). Conversely, on day 20 of lactation, plasma concentrations of Ile, Leu, Lys, Val, Cys, and total essential amino acids (**EAA**) increased with increasing SID Lys-to-NE ratio (linear; *P* < 0.05). In addition, plasma concentrations of Met, Phe, and Thr decreased then increased, while Gly increased then decreased (quadratic; *P *< 0.05) and Trp, Ala, and Asn tended to decrease then increase with increasing SID Lys-to-NE ratio (quadratic; *P* = 0.098, *P* = 0.056, and *P* = 0.053, respectively). The apparent utilization efficiencies of dietary AAs for milk protein production decreased with increasing SID Lys-to-NE ratio for all AAs in weeks 1, 2, and 3 of lactation (linear; *P *< 0.05; Table 6).

**Table 5.** Postabsorptive plasma concentrations of essential and nonessential AAs for primiparous sows fed one of five isoenergetic feeding programs that provided equally spaced and increasing SID Lys-to-NE ratios

	Diet^1^					SEM^2^	*P*-value^3^	
Item	2.85	3.51	4.18	4.84	5.51		Linear	Quadratic
**Day 4**								
No. of sows	10	10	11	11	11			
EAA^4^, μmol/L								
Arg	136	127	132	154	120	16	0.815	0.386
His	131	132	120	113	119	7	0.036	0.558
Ile	121	116	128	149	126	14	0.218	0.485
Leu	178	176	190	228	185	19	0.170	0.268
Lys	154	153	164	178	153	18	0.617	0.361
Met	46	43	42	43	42	3	0.342	0.568
Phe	85	78	85	95	85	7	0.267	0.811
Thr	192	157	169	161	148	15	0.031	0.610
Trp	54	51	47	59	51	4	0.932	0.734
Val	342	330	271	409	375	26	0.074	0.567
Total EAA	1,450	1,369	1,464	1,611	1,454	85	0.209	0.787
NEAA^5^, μmol/L								
Ala	592	590	516	510	496	51	0.038	0.741
Asn	61	57	54	52	53	4	0.024	0.348
Asp	15	17	16	16	13	1	0.118	0.109
Cys	15	9	12	16	15	3	0.441	0.272
Gln	618	645	566	491	529	40	0.003	0.768
Glu	193	189	164	153	170	14	0.041	0.171
Gly	950	957	907	837	782	73	0.015	0.498
Pro	272	255	242	264	244	19	0.330	0.609
Ser	145	130	136	146	133	7	0.637	0.787
Tyr	87	84	79	88	97	7	0.244	0.126
Total NEAA	2,938	2,931	2,688	2,626	2,620	166	0.024	0.742
**Day 12**								
No. of sows	10	9	11	10	11			
EAA^4^, μmol/L								
Arg	115	124	115	109	113	10	0.532	0.903
His	123	112	116	106	105	8	0.047	0.846
Ile	127	178	152	121	143	13	0.666	0.207
Leu	188	272	236	195	222	34	0.951	0.169
Lys	136	178	150	135	145	12	0.616	0.466
Met	37	29	31	32	31	3	0.187	0.115
Phe	84	98	86	79	88	6	0.511	0.820
Thr	152	132	136	123	140	13	0.294	0.144
Trp	55	53	46	53	51	4	0.479	0.251
Val	319	403	367	329	364	34	0.812	0.350
Total EAA	1,364	1,600	1,445	1,377	1,416	86	0.716	0.253
NEAA^5^, μmol/L								
Ala	441	364	332	325	327	29	0.002	0.051
Asn	56	46	47	43	44	4	0.011	0.153
Asp	15	13	12	12	11	1	0.021	0.789
Cys	19	30	19	26	22	5	0.704	0.396
Gln	545	418	457	456	430	28	0.020	0.112
Glu	200	198	159	162	154	16	0.006	0.556
Gly	809	679	673	764	672	61	0.267	0.392
Pro	213	202	194	195	203	12	0.381	0.195
Ser	142	143	121	124	122	8	0.008	0.747
Tyr	75	76	75	73	82	9	0.644	0.540
Total NEAA	2,674	2,319	2,122	2,261	2,044	211	0.037	0.239
**Day 20**								
No. of sows	10	10	10	10	10			
EAA^4^, μmol/L								
Arg	100	101	107	100	105	9	0.743	0.910
His	114	96	96	84	89	5	<0.001	0.101
Ile	122	122	133	143	151	12	0.043	0.704
Leu	178	186	200	229	235	17	0.005	0.842
Lys	121	122	118	145	159	12	0.006	0.134
Met	34	29	26	26	30	2	0.163	0.009
Phe	85	82	72	88	96	6	0.223	0.029
Thr	161	133	124	117	143	11	0.126	0.005
Trp	53	55	45	52	59	5	0.642	0.098
Val	279	288	325	363	382	29	0.002	0.807
Total EAA	1,244	1,214	1,268	1,345	1,434	77	0.029	0.301
NEAA^5^, μmol/L								
Ala	354	365	318	305	366	21	0.355	0.056
Asn	53	50	44	41	45	3	<0.001	0.053
Asp	11	12	11	12	10	1	0.113	0.258
Cys	16	9	16	26	20	3	0.019	0.607
Gln	465	503	399	396	360	32	0.001	0.504
Glu	153	166	152	155	132	10	0.103	0.960
Gly	704	768	762	672	549	50	0.029	0.010
Pro	177	194	179	179	187	7	0.840	0.792
Ser	131	122	124	115	106	5	<0.001	0.643
Tyr	65	66	59	68	74	5	0.283	0.201
Total NEAA	2,244	2,260	2,110	1,992	1,874	107	0.004	0.451

^1^Increasing SID Lys-to-NE ratios, g/Mcal.

^2^Largest value for standard error of means.

^3^Probability values for linear and quadratic contrasts.

^4^EAA: essential amino acids; Arg included as a conditionally EAA.

^5^NEAA: nonessential amino acids.

**Table 6.** Apparent utilization efficiency of dietary AAs for milk protein production between days 4 and 7 ± 1 (week 1), days 12 and 15 ± 1 (week 2), and days 20 and 23 ± 1 (week 3) of lactation in primiparous sows fed one of five isoenergetic feeding programs that provided equally spaced and increasing SID Lys-to-NE ratios

	Diet^1^					SEM^2^	*P*-value^3^	
Item	2.85	3.51	4.18	4.84	5.51		Linear	Quadratic
**Week 1 (days 4 to 7 ± 1)**								
No. of sows	10	10	11	11	11			
Arg	40.5	41.4	39.2	33.6	30.9	4.9	0.028	0.466
His	55.9	55.5	53.7	43.6	39.8	4.9	<0.001	0.327
Ile	53.4	55.5	56.5	46.4	43.8	4.7	0.022	0.168
Leu	53.4	55.7	57.5	47.0	44.9	4.7	0.037	0.165
Lys	73.9	71.1	68.1	53.9	49.5	6.1	0.001	0.465
Met	46.4	47.2	46.7	38.2	35.5	4.2	0.005	0.237
Met + Cys	50.2	51.9	53.0	43.2	40.7	4.3	0.015	0.155
Phe	39.3	40.4	40.8	32.8	31.3	3.4	0.009	0.202
Phe + Tyr	58.5	60.0	61.0	49.3	46.4	4.9	0.008	0.163
Thr	55.6	57.4	58.4	47.5	44.7	4.8	0.012	0.166
Val	46.1	48.1	49.0	40.6	38.7	4.2	0.044	0.206
N	70.1	69.0	70.4	54.0	50.3	5.4	<0.001	0.212
**Week 2 (days 12 to 15 ± 1)**								
No. of sows	10	9	11	10	11			
Arg	45.1	40.6	32.7	30.1	24.8	4.1	<0.001	0.749
His	56.2	49.3	41.1	37.0	31.8	4.7	<0.001	0.533
Ile	53.4	55.5	56.5	46.4	43.8	4.7	<0.001	0.168
Leu	51.2	47.1	42.0	39.0	35.4	4.4	<0.001	0.799
Lys	73.2	61.7	51.0	45.2	39.0	5.9	<0.001	0.356
Met	46.7	41.8	35.6	32.5	28.3	3.9	<0.001	0.684
Met + Cys	48.8	44.2	38.9	36.0	32.3	4.1	<0.001	0.735
Phe	38.6	34.9	30.4	27.4	24.9	3.3	<0.001	0.657
Phe + Tyr	56.5	50.7	44.6	40.9	36.8	4.7	<0.001	0.668
Thr	54.2	49.0	43.0	39.6	35.5	4.6	<0.001	0.707
Val	45.8	42.0	36.8	34.2	30.5	3.9	<0.001	0.807
N	63.4	54.0	48.2	42.7	39.5	5.0	<0.001	0.356
**Week 3 (days 20 to 23 ± 1)**								
No. of sows	10	10	10	10	10	10		
Arg	51.2	44.8	38.5	39.1	31.0	4.8	0.005	0.861
His	59.1	52.6	45.7	45.3	36.4	4.4	0.001	0.941
Ile	53.7	50.8	45.9	46.5	38.7	3.9	0.009	0.735
Leu	51.5	49.4	45.0	45.2	38.1	3.5	0.009	0.164
Lys	75.2	65.8	56.2	54.8	43.5	5.1	<0.001	0.837
Met	48.6	44.6	39.4	39.6	32.2	3.6	0.003	0.892
Met + Cys	49.1	46.5	42.1	42.3	35.3	3.4	0.006	0.702
Phe	39.3	36.8	33.1	32.7	27.5	2.8	0.004	0.822
Phe + Tyr	56.4	53.2	47.9	47.6	39.7	3.7	0.003	0.726
Thr	54.7	51.8	46.5	46.6	38.8	3.8	0.005	0.733
Val	47.0	44.5	40.4	41.0	34.2	3.5	0.013	0.730
N	59.2	55.2	48.7	45.0	38.4	3.2	<0.001	0.863

^1^Increasing SID Lys-to-NE ratios, g/Mcal.

^2^Largest value for standard error of means.

^3^Probability values for linear and quadratic contrasts.

## Discussion

The objective of the current study was to evaluate the SID Lys-to-NE ratios that minimize primiparous sow maternal N loss and maximize milk N output and piglet growth performance in each week of lactation. In the current study, maternal N retention (N retention − milk N output) was improved in each week of lactation and overall sow BW and BF depth losses were reduced when primiparous sows were fed increasing SID Lys-to-NE ratios. Additionally, fasted plasma concentrations of total NEAA decreased as the SID Lys-to-NE ratio increased within each week of lactation, which indicated reduced maternal protein mobilization (Reynolds et al., 1994). Conversely, total milk N output and overall piglet ADG were not influenced by SID Lys-to-NE ratio in any week of lactation, suggesting no difference in milk production in response to SID Lys-to-NE ratio. Therefore, it appears that lactating primiparous sows prioritize maternal fat and protein tissues, either by limiting mobilization or by allowing deposition, with increasing SID Lys (AA) supply, while excess, unbalanced AA was oxidized for energy to support maternal protein deposition rather than influencing milk N output or milk production. The supply of energy from the oxidation of AA-derived carbon skeletons could have been used to support either maternal protein deposition, since maternal N retention became positive with increasing SID Lys-to-NE ratio, or to minimize maternal lipid mobilization, since BF depth and BW losses were reduced with increasing SID Lys-to-NE ratio in the current study. Simultaneously, in the lowest SID Lys-to-NE ratio diets, Lys could have limited maternal protein deposition, particularly in week one of lactation when feed intake was low, since the apparent Lys utilization efficiency for milk production reached or exceeded the biological maximum (as defined by the NRC, 2012). In this scenario, however, it does not appear that (additional) energy generated from the unbalanced AA was used to limit maternal energy mobilization, since losses in BF depth were still the most extreme for sows fed the lowest SID Lys-to-NE ratios. In addition, as SID Lys-to-NE ratio increased, the apparent AA utilization efficiencies for milk production decreased concurrent with improvements in maternal nitrogen retention. The efficiency values were driven mainly by AA intake and AA output in milk, with estimated maternal AA retention contributing minimally, despite becoming positive at higher SID Lys-to-NE ratios and as lactation progressed. Indeed, the increasing SID Lys-to-NE ratios were achieved via inclusion of both L-Lys-HCl and soybean meal, which also increased the supply of CP and all other AA. With no change in milk AA output attributed to SID Lys-to-NE ratio, the apparent AA utilization efficiencies for milk production were thus reduced with greater SID Lys (AA) intakes. It has been shown previously that feeding reduced-CP diets supplemented with crystalline AA to improve AA balance (i.e., to reduce excess AA) increased the AA utilization efficiency for milk production (Huber et al., 2016; Zhang et al., 2019). Therefore, and particularly when the sow enters positive nitrogen balance, the apparent AA utilization efficiency calculation may not adequately capture maternal protein deposition.

Previous research has shown that primiparous sows can undergo extensive protein mobilization to maintain milk N output, suggesting that the maternal protein pool is more sensitive to dietary AA and energy supply than milk protein output (Clowes et al., 1998). Multiple research groups investigating increasing dietary CP and Lys contents have made similar observations for lactating sows, whereby increasing levels of dietary CP and/or Lys reduced body protein and BW loss (Dourmad et al., 1998; Hojgaard et al., 2019a; Strathe et al., 2020). It was also determined that increasing the inclusion of L-Lys in diets to increase Lys content, minimized body protein losses but did not impact body fat mobilization overall for sows from multiple parties (Hojgaard et al., 2019b). Additionally, sow body protein and fat contents were better maintained with greater supply of dietary energy compared to Lys (parities 1 to 5; Pedersen et al., 2019; Feyera et al., 2020). Conversely, in multiparous sows, increasing dietary CP or Lys supply increased milk protein content (Hojgaard et al., 2019a; Pedersen et al., 2019; Strathe et al., 2020) or milk N output (Strathe et al., 2017; Watzeck and Huber, 2024). Overall, primiparous sows appear to react differently to increasing SID Lys-to-NE ratios compared to multiparous sows, with increased amounts of dietary Lys required for first parity sows to maintain body protein pools during lactation, while maternal tissues are prioritized above (additional) milk production when surplus Lys (AAs) are available from the diet. The explanation for the diverging responses to SID Lys-to-NE ratios between primiparous and multiparous sows could simply be a matter of feed intake, whereby primiparous sows exhibit 10% lower feed intake versus multiparous sows as was demonstrated in the current study versus a previous study exploring optimal SID Lys-to-NE ratios for multiparous sows using the same genetics (Watzeck and Huber, 2024). Therefore, parity appears to have an impact dietary nitrogen and Lys utilization of lactating sows. Both parity and/or feed intake capacity should be taken into consideration when developing feeding programs for lactating sows in order to support optimal lactation performance for sows of all parities.

Nitrogen intake, N excretion, and N retention increased linearly as sows were fed increasing dietary SID Lys-to-NE ratios within each week of lactation. Nitrogen intake was increased with higher SID Lys-to-NE ratios due to the nature of the dietary blends, as the high-ratio diet had greater protein content compared to the low-ratio diet. This was to allow for an increased Lys content in the diet without using large amounts of crystalline AA and to maintain the AA-to-Lys ratios for the remaining EAA to ensure Lys was the first limiting AA in all the SID Lys-to-NE ratio blends. The increase in N intake and N retention has been observed in a previous study with similar experimental design but using multiparous sows (Watzeck and Huber, 2024) and in other work examining N utilization by lactating sows (Dourmad et al., 1998; Huber et al., 2015; Zhang et al., 2019). Moreover, in the current study, maternal nitrogen retention was negative for most sows in week 1, but became positive in weeks two and three of lactation. Such an increase in maternal N retention between early and peak lactation has also been observed in other studies (Pedersen et al., 2016; Zhang et al., 2019; Watzeck and Huber, 2024), and corresponds to the significant increase in lysine and energy requirements during the first week of lactation concurrent with low feed intake, followed by increased daily feed intakes as lactation progresses (Feyera and Theil, 2017).

Since milk N output and litter growth rate (milk yield) were not influenced by dietary SID Lys-to-NE ratio in the current study, maternal N retention was targeted as the outcome to evaluate the optimal SID Lys-to-NE ratio in each week of lactation. Moreover, the primiparous sow has not yet achieved mature BW, thus greater maternal N retention may improve ovarian function and second-litter characteristics (Clowes et al., 2003; De Rensis et al., 2005). In the current study, during the first week of lactation, maternal N retention remained in the linear phase and no optimal SID Lys-to-NE ratio was achieved. Therefore, at low feed intake for primiparous sows in week one of lactation, the optimal SID Lys-to-NE ratio was greater than 5.51 g SID Lys/Mcal NE (53 g SID Lys/day, based on the ADFI achieved in week 1). Conversely, when daily feed intake was increased by approximately 2 kg in the second and third weeks of lactation, maternal N retention was optimized at 4.74 and 4.84 g SID Lys/Mcal NE, respectively (68 and 79 g SID Lys/day, respectively). Alternatively, increasing dietary CP and Lys intakes in multiparous sows has been shown to positively impact litter ADG, with optimal growth achieved at 8.11 g SID Lys/kg of feed (estimated 52 g SID Lys/day) and a SID Lys-to-NE ratio of 4.3 g SID Lys/Mcal NE (estimated 64 g SID Lys/day; Hojgaard et al, 2019b; Watzeck and Huber, 2024). Thus, the SID Lys-to-NE ratios necessary to optimize milk yield in multiparous sows are relatively less than those found in the current study to optimize maternal nitrogen retention of primiparous sows. The optimum SID Lys-to-NE ratios determined in the current study were also greater than current industry feeding standards (~3.9 g SID Lys/Mcal NE), which could place primiparous sows at a disadvantage in terms of maternal protein loss during lactation. Finally, in weeks two and three of lactation, the optimal SID Lys-to-NE ratios were rather similar for maternal N retention, indicating that primiparous sows would benefit most from a high ratio during the first week of lactation but could receive a relatively constant ratio thereafter.

## Conclusion

In conclusion, increasing SID Lys-to-NE ratios for primiparous sows increased maternal N retention and helped to maintain sow BW and BF depth, without influencing milk N output or piglet growth performance. Therefore, it is possible to create dynamic feeding programs by utilizing two extreme diets to deliver optimized SID Lys-to-NE ratios throughout lactation for primiparous sows, which could allow for a reduction in maternal tissue mobilization with the potential to improve subsequent reproductive performance and sow longevity.

## Abbreviations:

AA: amino acids
ADG: average daily gain
BF: backfat
BW: body weight
CP: crude protein
EAA: essential amino acids
LBL: linear broken-line
NE: net energy; N, nitrogen
NEAA: nonessential amino acids
SID: standardized ileal digestible

## References

[CIT0001] AOAC. 2005. Official methods of analysis of AOAC International. 18th ed. Rockville (MD): Assoc. Off. Anal. Chem Int.

[CIT0002] AOCS. 2017. Official methods and recommended practices of the AOCS. 7th ed. Urbana (IL): AOCS.

[CIT0003] Banton, S., J. G. Pezzali, A. Verbrugghe, M. Bakovic, K. M. Wood, and A. K. Shoveller. 2021. Addition of dietary methionine but not dietary taurine or methyl donors/receivers to a grain-free diet increases postprandial homocysteine concentrations in adult dogs. J. Anim. Sci. 99:skab223. doi: https://doi.org/10.1093/jas/skab223 34333630 PMC8420682

[CIT0004] Bidlingmeyer, B. A., S. A. Cohen, and T. L. Tarvin. 1984. Rapid analysis of amino acids using pre-column derivatization. J. Chromatogr. B. Biomed. Sci. Appl. 336:93–104. doi: https://doi.org/10.1016/s0378-4347(00)85133-6 6396315

[CIT0005] Canadian Council on Animal Care (CCAC). 2009. Guidelines on: the care and use of farm animals in research, teaching and testing. Ottawa (ON): Canadian Council on Animal Care (CCAC).

[CIT0007] Clowes, E. J., I. H. Williams, V. E. Baracos, J. R. Pluske, A. C. Cegielski, L. J. Zak, and F. X. Aherne. 1998. Feeding lactating primiparous sows to establish three divergent metabolic states: II. Effect on nitrogen partitioning and skeletal muscle composition. J. Anim. Sci. 76:1154–1164. doi: https://doi.org/10.2527/1998.7641154x 9581940

[CIT0006] Clowes, E. J., F. X. Aherne, G. R. Foxcroft, and V. E. Baracos. 2003. Selective protein loss in lactating sows is associated with reduced litter growth and ovarian function. J. Anim. Sci. 81:753–764. doi: https://doi.org/10.2527/2003.813753x 12661656

[CIT0008] Crosbie, M., C. Zhu, A. K. Shoveller, and L. Huber. 2020. Standardized ileal digestible amino acids and net energy contents in full fat and defatted black soldier fly larvae meals (*Hermetia illucens*) fed to growing pigs. Transl Anim Sci 4:txaa104. doi: https://doi.org/10.1093/tas/txaa104 32734146 PMC7381833

[CIT0009] De Rensis, F., M. Gherpelli, P. Superchi, and R. N. Kirkwood. 2005. Relationships between backfat depth and plasma leptin during lactation and sow reproductive performance after weaning. Anim. Reprod. Sci. 90:95–100. doi: https://doi.org/10.1016/j.anireprosci.2005.01.017 16257599

[CIT0010] Dourmad, J. Y., J. Noblet, and M. Étienne. 1998. Effect of protein and lysine supply on performance, nitrogen balance, and body composition changes of sows during lactation. J. Anim. Sci. 76:542–550. doi: https://doi.org/10.2527/1998.762542x 9498364

[CIT0012] Feyera, T., and P. K. Theil. 2017. Energy and lysine requirements and balances of sows during transition and lactation: a factorial approach. Livestock Sci. 201:50–57. doi: https://doi.org/10.1016/j.livsci.2017.05.001

[CIT0011] Feyera, T., U. Krogh, T. Hinrichsen, T. S. Bruun, and P. K. Theil. 2020. A two-component feeding strategy with high supply of energy and lysine ensured a high milk yield, minimal mobilization and improved feed efficiency of lactating sows. Livestock Sci. 240:104162. doi: https://doi.org/10.1016/j.livsci.2020.104162

[CIT0013] Gonçalves, M. A. D., N. M. Bello, S. S. Dritz, M. D. Tokach, J. M. DeRouchey, J. C. Woodworth, and R. D. Goodband. 2016. An update on modeling dose–response relationships: Accounting for correlated data structure and heterogeneous error variance in linear and nonlinear mixed models. J. Anim. Sci. 94:1940–1950. doi: https://doi.org/10.2527/jas.2015-0106 27285692

[CIT0014] Hojgaard, C. K., T. S. Bruun, and P. K. Theil. 2019a. Optimal crude protein in diets supplemented with crystalline amino acids fed to high-yielding lactating sows. J. Anim. Sci. 97:3399–3414. doi: https://doi.org/10.1093/jas/skz200 31190051 PMC6667265

[CIT0015] Hojgaard, C. K., T. S. Bruun, and P. K. Theil. 2019b. Optimal lysine in diets for high-yielding lactating sows. J. Anim. Sci. 97:4268–4281. doi: https://doi.org/10.1093/jas/skz286 31504612 PMC6776283

[CIT0016] Hoving, L. L., N. M. Soede, E. A. M. Graat, H. Feitsma, and B. Kemp. 2011. Reproductive performance of second parity sows: Relations with subsequent reproduction. Livestock Sci. 140:124–130. doi: https://doi.org/10.1016/j.livsci.2011.02.019

[CIT0017] Huber, L., C. F. M. de Lange, U. Krogh, D. Chamberlin, and N. L. Trottier. 2015. Impact of feeding reduced crude protein diets to lactating sows on nitrogen utilization. J. Anim. Sci. 93:5254–5264. doi: https://doi.org/10.2527/jas.2015-9382 26641045

[CIT0018] Huber, L., C. F. M. de Lange, C. W. Ernst, U. Krogh, and N. L. Trottier. 2016. Impact of improving dietary amino acid balance for lactating sows on efficiency of dietary amino acid utilization and transcript abundance of genes encoding lysine transporters in mammary tissues. J. Anim. Sci. 94:4654–4665. doi: https://doi.org/10.2527/jas.2016-0697 27898953

[CIT0019] Möhn, S., and C. F. M. de Lange. 1998. The effect of body weight on the upper limit to protein deposition in a defined population of growing gilts. J. Anim. Sci. 76:124–133. doi: https://doi.org/10.2527/1998.761124x 9464893

[CIT0020] NRC. 2012. Nutrient requirements of swine. 11th rev. ed. Washington (DC): Natl. Acad. Press.

[CIT0021] Pedersen, T. F., T. S. Bruun, T. Feyera, U. K. Larsen, and P. K. Theil. 2016. A two-diet feeding regime for lactating sows reduced nutrient deficiency in early lactation and improved milk yield. Livestock Sci. 191:165–173. doi: https://doi.org/10.1016/j.livsci.2016.08.004

[CIT0022] Pedersen, T. F., C. Y. Chang, N. L. Trottier, T. S. Bruun, and P. K. Theil. 2019. Effect of dietary protein intake on energy utilization and feed efficiency of lactating sows. J. Anim. Sci. 97:779–793. doi: https://doi.org/10.1093/jas/sky462 30535080 PMC6358224

[CIT0023] Reynolds, C. K., D. L. Harmon, R. L. Prior, and H. F. Tyrrell. 1994. Effects of mesenteric vein L-alanine infusion on liver metabolism of organic acids by beef heifers fed diets differing in forage: concentrate ratio. J. Anim. Sci. 72:3196–3206. doi: https://doi.org/10.2527/1994.72123196x 7759370

[CIT0024] Schenkel, A. C., M. L. Bernardi, F. P. Bortolozzo, and I. Wentz. 2010. Body reserve mobilization during lactation in first parity sows and its effect on second litter size. Livestock Sci. 132:165–172. doi: https://doi.org/10.1016/j.livsci.2010.06.002

[CIT0025] Strathe, A. V., T. S. Bruun, N. Geertsen, J. -E. Zerrahn, and C. F. Hansen. 2017. Increased dietary protein levels during lactation improved sow and litter performance. Anim. Feed Sci. Technol. 232:169–181. doi: https://doi.org/10.1016/j.anifeedsci.2017.08.015

[CIT0026] Strathe, A. V., T. S. Bruun, A. -H. Tauson, P. K. Theil, and C. F. Hansen. 2020. Increased dietary protein for lactating sows affects body composition, blood metabolites and milk production. Animal 14:285–294. doi: https://doi.org/10.1017/S1751731119001678 31368423

[CIT0027] Tokach, M. D., M. B. Menegat, K. M. Gourley, and R. D. Goodband. 2019. Review: Nutrient requirements of the modern high-producing lactating sow, with an emphasis on amino acid requirements. Animal 13:2967–2977. doi: https://doi.org/10.1017/S1751731119001253 31199216

[CIT0028] Watzeck, M. C., and L. Huber. 2024. The standardized ileal digestible lysine-to-net energy ratio in the diets of sows to optimize milk nitrogen retention is dynamic during lactation. J. Anim. Sci. 102:skae094. doi: https://doi.org/10.1093/jas/skae094 38558239 PMC11056879

[CIT0029] Zhang, S., M. Qiao, and N. L. Trottier. 2019. Feeding a reduced protein diet with a near ideal amino acid profile improves amino acid efficiency and nitrogen utilization for milk production in sows. J. Anim. Sci. 97:3882–3897. doi: https://doi.org/10.1093/jas/skz220 31394569 PMC6735961

[CIT0030] Zhu, C., M. Rademacher, and C. F. M. de Lange. 2005. Increasing dietary pectin level reduces utilization of digestible threonine intake, but not lysine intake, for body protein deposition in growing pigs. J. Anim. Sci. 83:1044–1053. doi: https://doi.org/10.2527/2005.8351044x 15827249

